# Profiling of hepatic clearance pathways of Pittsburgh compound B and human liver cytochrome p450 phenotyping

**DOI:** 10.1186/2191-219X-3-10

**Published:** 2013-02-14

**Authors:** Anne Van Vlaslaer, Russell J Mortishire-Smith, Claire Mackie, Xavier Langlois, Mark E Schmidt

**Affiliations:** 1 C.R.E.A.Te, Janssen Pharmaceutical Companies of Johnson & Johnson, Turnhoutseweg 30, B-2340, Beerse, Belgium; 2 Project Management Office, Janssen Pharmaceutical Companies of Johnson & Johnson, Turnhoutseweg 30, B-2340, Beerse, Belgium; 3 Neuroscience TA, Janssen Pharmaceutical Companies of Johnson & Johnson, Turnhoutseweg 30, B-2340, Beerse, Belgium; 4 Experimental Medicine, Janssen Research and Development, Division of Janssen Pharmaceutica, NV Turnhoutseweg 30, B-2340, Beerse, Belgium

**Keywords:** Amyloid, Pittsburgh compound B, Drug metabolism, Hepatic clearance, P450 phenotyping

## Abstract

**Background:**

^11^C-PiB has been developed as a positron-emission tomography (PET) ligand for evaluating fibrillar β-amyloid (Aβ) in the human brain. The ligand is rapidly metabolized, with approximately 10% of intact tracer remaining 30 min after injection. When ^11^C-PiB is used as a treatment endpoint in intervention studies for Alzheimer’s disease (AD), a concern is whether the clearance of the tracer changes from one scan to the next, increasing within subject variability in the PET signal. Subjects enrolled in AD trials may start or stop medications that inhibit or induce xenobiotic metabolizing enzymes such as the cytochrome P450 (CYP) isozymes.

**Findings:**

We conducted CYP phenotyping in recombinantly expressed systems, and in human liver microsomes, to evaluate CYP isozyme contributions to the metabolism of PiB (carrier) and profiled microsomal and hepatocyte incubations for metabolites. The metabolism of PiB appears to be polyzymic, with direct conjugation via UDP-glucuronosyltransferases (UGTs) also occurring.

**Conclusion:**

It is unlikely that CYP inhibition or induction will significantly influence the clearance of ^11^C-PiB.

## Findings

### Background

The thioflavin T derivative, *N*-methyl-^11^C]2-(4^′^methylaminophenyl)-6 hydroxybenzothiazole (^11^C-PiB), has been developed as a positron-emission tomography (PET) ligand for evaluating fibrillar β-amyloid (Aβ) in the human brain. Retention of ^11^C-PiB in the cortex correlates with the distribution of insoluble deposits of Aβ [1] and has been investigated extensively as a biomarker for Alzheimer’s disease (AD) [2] and used as an endpoint in interventional trials [3,4]. ^11^C-PiB is rapidly cleared from the blood, with approximately 10% of intact tracer remaining 30 min after injection [5]. Test-retest studies conducted hours to weeks apart have reported interscan differences of less than 10% [6]. These test-retest estimates may not predict the variability in measurement over longer periods of time, such as months to years.

A critical issue is the possible changes in clearance of the tracer as this could alter the input function into the brain. Metabolism of a tracer that depends primarily on one of the cytochrome P450 (CYP) isoenzymes could be vulnerable to inhibition by particular medications. Subjects enrolled in AD trials may start or stop a number of medications that inhibit or induce CYP enzymes such as statins, well-known potent inhibitors of the CYP3A isoenzymes, or experience conditions that could affect CYP expression [7]. The metabolism of the serotonin 5HT1A receptor PET ligand FCWAY is significantly reduced by pretreatment with disulfuram, a potent inhibitor of CYP2E1. The ratio of specific distribution volume of tracer in target rich to target poor regions increased twofold after pretreatment with disulfuram [8]. Such a change would confound measurement of change in PiB signal as an indicator of disease progression and potentially obscure detection of treatment effects using PiB as an endpoint. Current practice in acquiring PiB PET scans does not include any blood sampling, so changes in tracer clearance from one scan to the next would not be detected. We wished to determine the metabolic pathway for PiB to explore whether peripheral clearance of ^11^C-PiB could be susceptible to CYP inhibition or induction - specifically, whether the metabolism of PiB via the most abundant CYPs is mono- or polyzymic, and whether direct conjugation of PiB is a significant clearance pathway. While these *in vitro* systems are necessarily reductionist in comparison with the *in vivo* systems, they are generally agreed to provide valuable insight into likely *in vivo* outcomes.

### Materials and methods

Cryopreserved human (male and female), male Beagle dog, male Sprague–Dawley rat, and male Swiss mouse hepatocytes were purchased from In Vitro Technologies (In Vitro Technologies, Baltimore, MD, USA). Sulfaphenazole and quinidine were purchased from Sigma (St. Louis, MO, USA). Alpha-naphthoflavone was purchased from Merck KGaA (Darmstadt, Germany). Benzylphenobarbital and ketoconazole were synthesized in-house. PiB was purchased from ABX GmbH (Radeberg, Germany). Diagnostic P450 inhibitors and test compounds were supplied as dry powder and dissolved in 100% dimethylsulfoxide (DMSO). All other materials were of analytical or higher grade and used without further purification. Cytochrome P450 isoforms 1A2, 2C9, 2C19, 2D6, and 3A4 were obtained as singly expressed enzymes (*Baculovirus* transfected insect cell system, rhCYPs) from BD Gentest (Woburn, MA, USA). Human liver microsomes (HLM) were obtained from BD Gentest (Woburn, MA, USA) as a pool from 50 donors, lot 01220.

#### Incubations with recombinantly expressed cytochrome P450s

The assay mixtures (final incubation volume of 125 μL) in 0.255 M phosphate buffer with 0.575% (*w*/*v*) KCl (pH 7.4) contained PiB (1.0 μM), 2.0 mM nicotinamide-adenine dinucleotide phosphate (reduced form) (NADPH), and a recombinantly expressed cytochrome P450 enzyme preparation at 100 pmol/mL. After a 10 min preincubation at 37°C, the reaction was initiated by addition of 2.0 mM NADPH and incubated for 15 and 60 min. The reactions were terminated by the addition of two volumes of DMSO. Samples were subsequently centrifuged at 4,000 rpm for 10 min. From the resulting supernatant, 10 μL was analysed by liquid chromatography/tandem mass spectrometry (LC-MS/MS). Control experiments were performed by substituting the active enzyme preparation by insect cell preparations containing no rhCYP.

#### Human liver microsomal metabolism in the absence and presence of cytochrome P450 diagnostic inhibitors

Assay mixtures (final incubation volume of 125 μL) in 0.255 M phosphate buffer with 0.575% (*w*/*v*) KCl (pH 7.4) contained PiB (1.0 μM), an NADPH regenerating system (consisting of 1.65 mM glucose-6-phosphate, 0.125 U glucose-6-phosphate dehydrogenase, 0.1 mM NADP, and 5.0 mM magnesium chloride), 1.0 mg/mL of pooled human liver microsomes, and alpha-naphthoflavone (3 μM), sulfaphenazole (10 μM), benzylphenobarbital (1 μM), quinidine (3 μM), or ketoconazole (1 μM), and inhibitors of cytochromes P450 1A2, 2C9, 2C19, 2D6, and 3A4, respectively. After a 10 min preincubation at 37°C, the reaction was initiated by addition of 0.15 mM NADP and further incubated for either 15 or 60 min. The reactions were terminated by the addition of two volumes DMSO. Samples were subsequently centrifuged at 4,000 rpm for 10 min. From the resulting supernatant, 10 μL was analyzed by LC-MS/MS.

#### Hepatocyte incubations

PiB was incubated in 24-well plates at a final concentration of 1.0 μM for 0, 15, and 60 min, with hepatocyte cell cultures (1 million viable cells/mL), after which the incubations were quenched with two volumes of acetonitrile.

#### LC-MS/MS detection

Quantification was performed on a TSQ-Vantage instrument (Thermofisher Scientific, Bremen, Germany), fitted with an electrospray ionization source. The S-lens voltage was set to 78 V, and the intensity of two transitions (257 > 242 at a collision energy (CE) = 27 V; 257 > 215, CE = 36 V) were measured during the experiment. Elution was performed on a Waters XBridge C18 column (3 × 2.1 mm, 2.5 μm; Waters, Boston MA, USA). Solvents A (0.1% formic acid in water) and B (0.1% formic acid in acetonitrile) were used at a flow rate of 500 μL/min. The gradient employed was solvent A 95% as the start condition followed by a linear ramp of organic phase concentration from 5% B to 95% B over 1.3 min. The final ratio was held for a further 0.6 min and then reduced again to 5% B over 0.05 min. Data was processed using QuickQuan (Thermofisher Scientific, Bremen, Germany).

Qualitative high resolution LC/MS data was acquired on a Waters Premier hybrid quadrupole time-of-flight (QToF) mass spectrometer (Waters, Boston MA, USA) equipped with a liquid chromatography system, using a 10 min gradient method and an Acquity UPLC BEH C18 (2.1 × 100 mm, 1.7 μm) column (Waters, Boston MA). The QToF Premier operated with a cone voltage of 30 eV and collision energy of 30 eV. The gradient employed was 95% solvent A (25 mM ammonium acetate, pH = 9) as the start condition followed by a linear ramp of organic phase concentration from 5% B (25 mM ammonium acetate/MeOH/acetonitrile 10/10/80) to 95% B over 6.5 min. The final ratio was held for 2 min and then reduced again to 5% B over 1 min. Interpretation of data was performed with Metabolynx software (Waters, Boston, MA, USA) [9].

### Results

**Figure 1:**
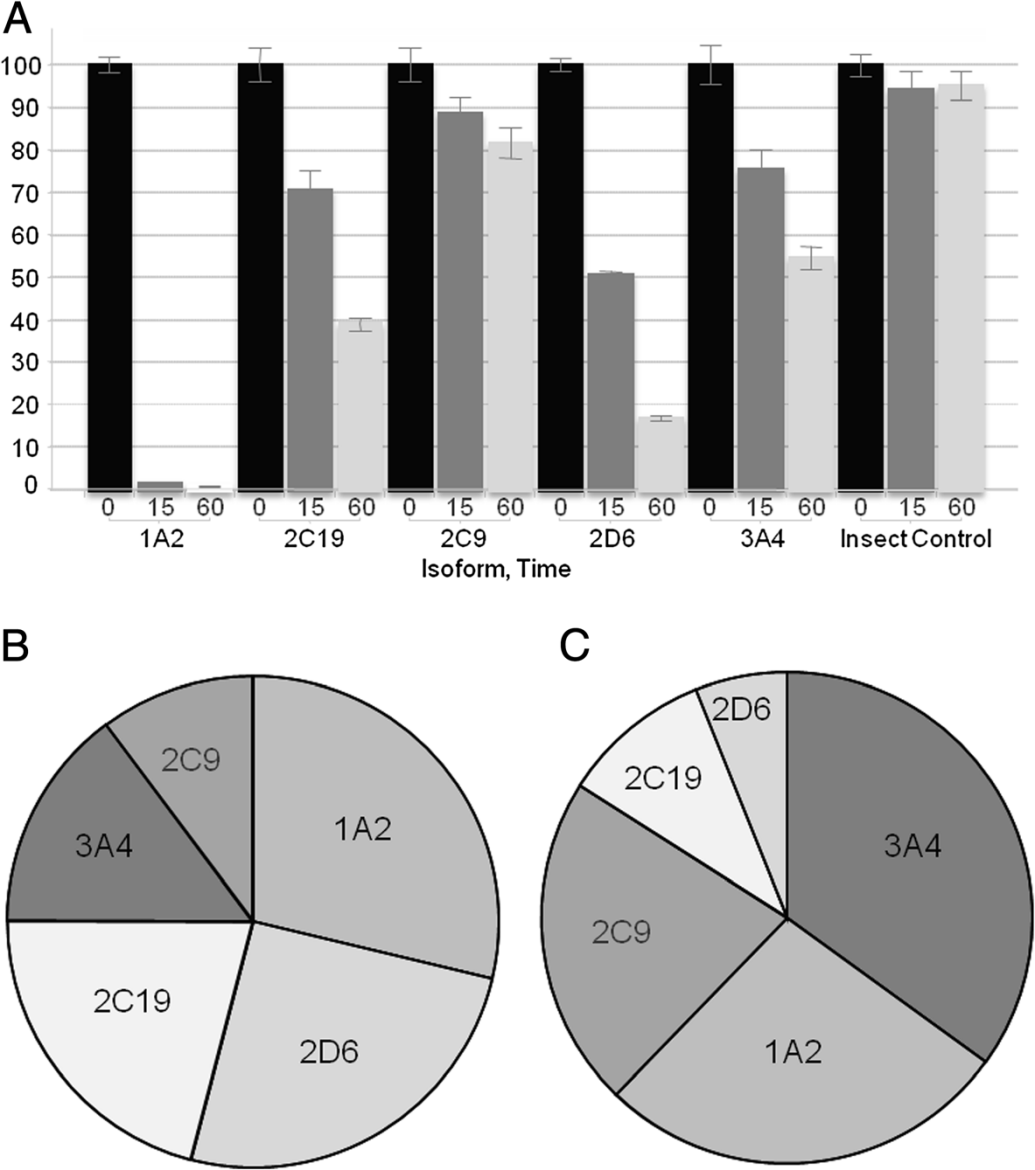
**Percentage of PiB remaining after incubation in HLM and relative contributions to turnover. (A)** % PiB remaining after 0 (black), 15 (dark gray) and 60 min (light gray) incubations at 1 μM substrate concentration with rhCYPs 1A2, 2C9, 2C19, 2D6, and 3A4. **(B)** Relative contributions to turnover of PiB based on unscaled rhCYP data. **(C)** Relative contributions to turnover of PiB after scaling for the relative abundances of CYPs in human liver, according to Rodrigues [10].

Based on the observed turnovers, PiB is a substrate for all five of the major human CYPs, with rhCYP1A2 dominating. Each incubation only contains a single CYP at an effective abundance of 100%. Scaling for the approximate relative abundances of the major five CYP isoforms in human liver [10] indicates that PiB is likely to be primarily metabolized by CYPs 3A4, 1A2, and 2C9, with only minor contributions from 2C19 and 2D6 (Figure 1A,B).

**Figure 2:**
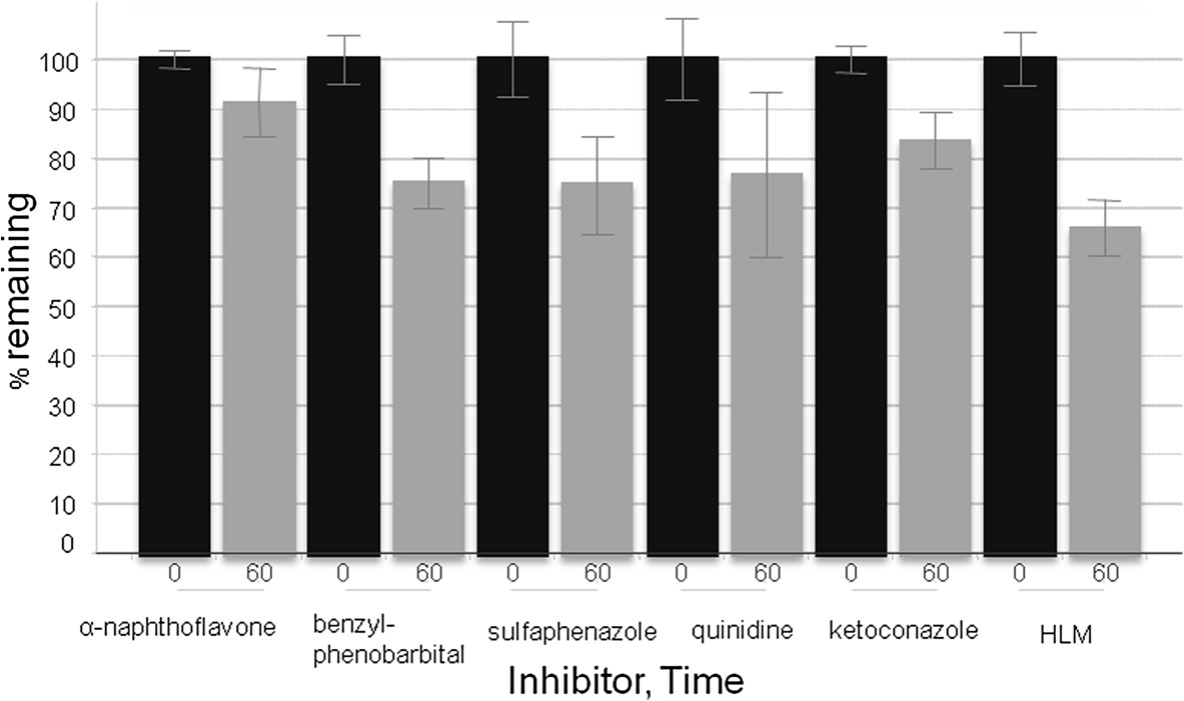
**Percentage of PiB remaining after 60 min incubations in HLM.** Percentage of PiB remaining after 0 min (black) and 60 min (dark gray) incubations in HLM at 1 μM substrate concentration with no inhibition (two rightmost bars), or in the presence of CYP-specific chemical inhibitors.

The turnover of PiB in human liver microsomes was approximately 35% after 60 min. A significant reduction in turnover was observed with inhibitors of CYPs 1A2 and 3A4, and a reduction in turnover is also apparent for CYPs 2C19, 2C9, and 2D6 (Figure 2).

**Figure 3:**
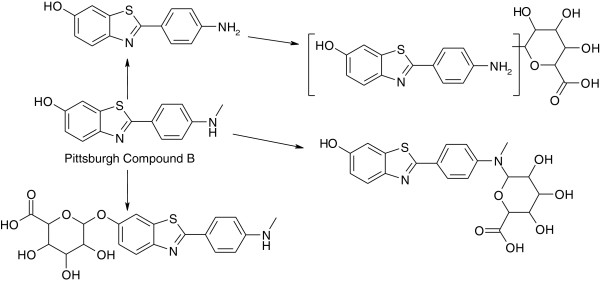
Metabolites identified in incubations with human liver microsomes and hepatocytes.

The HLM incubations from the phenotyping experiments and the separate incubations performed with human, rat, dog, and mouse hepatocytes were profiled for PiB metabolites. The only drug-related species detected in HLM was the desmethyl metabolite. Additionally present in human hepatocyte incubations were two glucuronide conjugates, assumed to be formed by direct glucuronidation of the phenolic hydroxyl group and of the secondary amine. A combination demethylation/glucuronidation metabolite was also detected, but it was not possible to assign the glucuronidation site for this metabolite (Figure 3).

**Figure 4:**
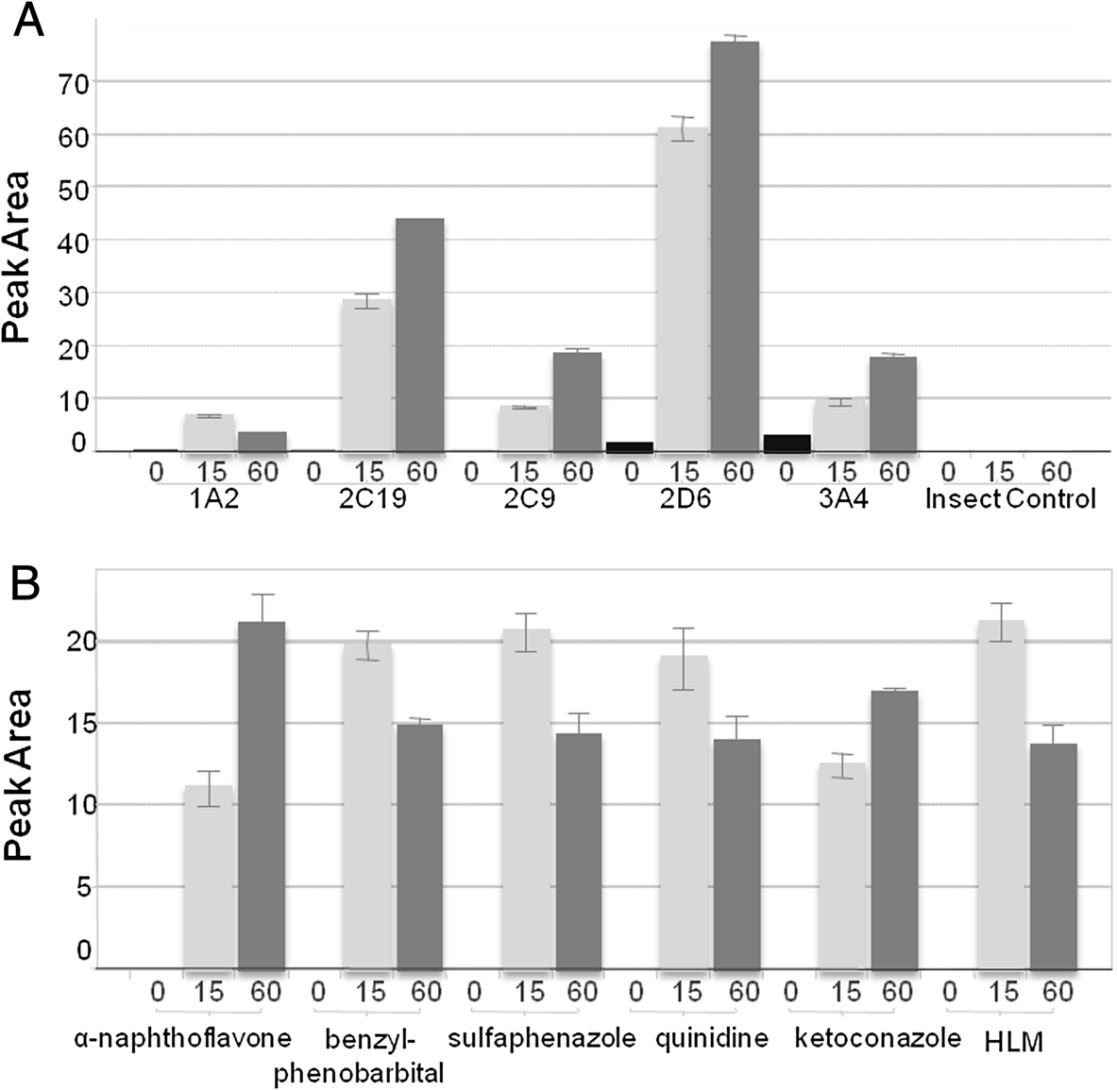
**Peak areas observed for the desmethyl metabolite. (A)** RhCYP incubations. **(B)** Incubations with HLM in the presence or absence of chemical inhibitors.

In rhCYP incubations, the desmethyl metabolite was the only drug-related species observed. Despite high turnover with 1A2 (and 3A4), only low levels of the desmethyl metabolite were present. In the HLM incubations with inhibitors of 1A2 and 3A4, concentrations of the desmethyl metabolite increased over time, while with 2C19, 2C9, and 2D6, concentrations were reduced at 60 min compared with 15 min (Figure 4). These data are together consistent with a two-step pathway in which all isoforms are able to demethylate, but 1A2 and 3A4 can then metabolize the desmethyl metabolite to a secondary product not detectable within the limits of detection of the qualitative LC/MS method used.

### Discussion

The results of experiments using *in vitro* human systems indicate that PiB is metabolized via both oxidative and conjugative mechanisms. The dominant routes of metabolism are N-demethylation and glucuronidation. The metabolism of PiB appears to be polyzymic, with at least two of the major human CYPs (1A2 and 3A4), contributing to metabolism, together with UDP-glucuronosyltransferase (UGT) involvement.

These results assume that the principal pathways observed in these *in vitro* human metabolizing systems translate to *in vivo*. *In vivo* drug metabolism is typically found to be more, not less complex than that observed *in vitro* and one metabolite known to be formed in man, a sulfate conjugate [11], was not observed in these experiments. Moreover, the absolute contributions of oxidation and conjugation to clearance cannot be measured without authentic standards. A fuller understanding of the enzyme kinetics involved in biotransformation of PiB would afford improved predictions of its *in vivo* pharmacokinetics and susceptibility to drug-drug interactions. Even so, the multiple pathways determined here to be involved in the metabolic clearance of PiB reduce the likelihood that drug-drug interactions are a source of variability in quantitative measurement of ^11^C-PiB PET.

## Competing interests

All authors are full time employees of Janssen Pharmaceutica, N.V. None of the authors have financial interests in GE Healthcare, the commercial license holder of the ^11^C-PiB technology; nor with the University of Pittsburgh, the licensor of the technology. Janssen Pharmaceutica, N.V. is engaged in the discovery and development of new treatments for Alzheimer’s disease. Availability of reliable methods of longitudinal measurement of brain fibrillar amyloid using PET may be of critical importance for interventional studies of Alzheimer’s disease.

## Authors’ contributions

AVV conducted all *in vitro* assays for CYP phenotyping, microsomal and hepatocyte incubations, and drafted the manuscript. RMS selected the *in vitro* assays, reviewed and approved all results, and provided the interpretation of metabolic pathways. CM drafted the research plan. XL contributed to experimental design and conducted supportive studies. MES conceived the project and assisted in the interpretation of results. All authors contributed to the writing and review of the reports and have read and approved the manuscript.
